# PSMD8 can serve as potential biomarker and therapeutic target of the PSMD family in ovarian cancer: based on bioinformatics analysis and in vitro validation

**DOI:** 10.1186/s12885-023-11017-8

**Published:** 2023-06-22

**Authors:** Xiao Li, Xinru Li, Yuexin Hu, Ouxuan Liu, Yuxuan Wang, Siting Li, Qing Yang, Bei Lin

**Affiliations:** 1 Department of Obstetrics and Gynecology, Shengjing Hospital Affiliated to China Medical University, No. 36, Sanhao Street, Heping District, Shenyang, 110004 People’s Republic of China; 2 Key Laboratory of Obstetrics and Gynecology of Higher Education of Liaoning Province, Shenyang, China

**Keywords:** PSMD8, Poor prognosis, Malignant behavior, Ovarian cancer

## Abstract

**Background:**

The ubiquity-proteasome system is an indispensable mechanism for regulating intracellular protein degradation, thereby affecting human antigen processing, signal transduction, and cell cycle regulation. We used bioinformatics database to predict the expression and related roles of all members of the *PSMD* family in ovarian cancer. Our findings may provide a theoretical basis for early diagnosis, prognostic assessment, and targeted therapy of ovarian cancer.

**Methods:**

GEPIA, cBioPortal, and Kaplan–Meier Plotter databases were used to analyze the mRNA expression levels, gene variation, and prognostic value of *PSMD* family members in ovarian cancer. *PSMD8* was identified as the member with the best prognostic value. The TISIDB database was used to analyze the correlation between *PSMD8* and immunity, and the role of PSMD8 in ovarian cancer tissue was verified by immunohistochemical experiments. The relationship of PSMD8 expression with clinicopathological parameters and survival outcomes of ovarian cancer patients was analyzed. The effects of PSMD8 on malignant biological behaviors of invasion, migration, and proliferation of ovarian cancer cells were studied by in vitro experiments.

**Results:**

The expression levels of *PSMD8/14* mRNA in ovarian cancer tissues were significantly higher than those in normal ovarian tissues, and the expression levels of *PSMD2/3/4/5/8/11/12/14* mRNA were associated with prognosis. Up-regulation of *PSMD4/8/14* mRNA expression was associated with poor OS, and the up-regulation of *PSMD2/3/5/8* mRNA expression was associated with poor PFS in patients with ovarian serous carcinomas. Gene function and enrichment analysis showed that *PSMD8* is mainly involved in biological processes such as energy metabolism, DNA replication, and protein synthesis. Immunohistochemical experiments showed that PSMD8 was mainly expressed in the cytoplasm and the expression level was correlated with FIGO stage. Patients with high PSMD8 expression had poor prognosis. Overexpression of PSMD8 significantly enhanced the proliferation, migration, and invasion abilities in ovarian cancer cells.

**Conclusion:**

We observed different degrees of abnormal expression of members of *PSMD* family in ovarian cancer. Among these, PSMD8 was significantly overexpressed in ovarian malignant tissue, and was associated with poor prognosis. *PSMDs*, especially *PSMD8*, can serve as potential diagnostic and prognostic biomarkers and therapeutic targets in ovarian cancer.

**Supplementary Information:**

The online version contains supplementary material available at 10.1186/s12885-023-11017-8.

## Background

Ovarian cancer is one of the most common malignant tumors of the female reproductive system. The currently established treatment methods include surgery, platinum and paclitaxel-based chemotherapy. Although the widespread use of molecular targeted therapies have helped improve the survival rate of some ovarian cancer patients, the five-year survival rates have remained within the range of 25%–30% [1–3]. Therefore, identification of efficient biomarkers is a key imperative to provide a basis for early diagnosis, targeted therapy, and precise prognostic assessment.

The ubiquitin–proteasome system is mainly composed of ubiquitin, ubiquitin-activating enzymes(E1s), ubiquitin-conjugating enzymes(E2s), ubiquitin ligases(E3s), 26S proteasome and deubiquitinating enzymes, which promote degradation of damaged proteins, and regulate growth and stress responses. The ubiquitin–proteasome system is an indispensable mechanism for regulating intracellular protein degradation, thereby affecting human antigen processing, signal transduction, and cell cycle regulation [4]. The 26S proteasome consists of a proteolytically active cylindrical particle (20S proteasome) and one or two ATPase-containing complexes (called 19S cap complexes), which are divided into ATPase subunits (PSMC 1 ~ 6) and non-ATPase subunits (PSMD1-14) [5–8]. Recent studies have shown that dysfunction of the ubiquitin–proteasome system can lead to up-regulation and/or down-regulation of *PSMDs* genes, and abnormal gene expression is often associated with oncogenes and tumor suppressor genes that regulate tumors [9–11]. As ATP independent molecules, PSMDs complete biological functions without external energy input and obvious conformational changes, and play an important role in a variety of cancers, which attracted our attention. The expression of the *PSMDs* has been first studied in breast cancer and bladder cancer, but its carcinogenic or anticancer effect in ovarian cancer has not been reported. Therefore, in this study, we evaluated the role of *PSMD* family in ovarian cancer. Our findings may provide a theoretical basis for the early diagnosis, prognostic assessment, and targeted therapy of ovarian cancer.

## Materials and methods

### GEPIA dataset analysis

The GEPIA database (http://gepia.cancer-pku.cn/) [12] contains data pertaining to 9736 tumor samples and 8587 normal samples, based on the UCSC Xena data. We used the GEPIA dataset to validate *PSMDs* in ovarian cancer and assessed the differences in mRNA expressions between ovarian cancer tissues and adjacent normal tissues. *P* < 0.05 was considered indicative of statistical significance.

### TISIDB analysis

The TISIDB database (http://cis.hku.hk/TISIDB) [13] is a resource for immunology data including high-throughput screening data, tumor immune-related genes, molecular profiles, and paracancerous multi-omics data. The database can be used to assess the correlation of genes with lymphocyte subsets, immunomodulators, chemokines, etc. We used this database to analyze the relationship between *PSMDs* expression and clinical stage in ovarian cancer, and to explore the correlation of *PSMD8* with lymphocytes and immunomodulators.

### TCGA and cBioPortal analysis

cBioPortal is an open database based on the TCGA database (www.cbioportal.org) [14] for interactive exploration of multiple cancer genomics datasets. The database contains data pertaining to DNA copies, DNA methylation, mRNA and microRNA expression, non- synonymous mutation, and other data. In this study, we used this database to analyze the gene variation of *PSMDs*, and evaluate co-expression correlation between members of *PSMDs*.

### Kaplan–Meier Plotter analysis

As a biomarker evaluation tool, the Kaplan–Meier plotter (http://kmplot.com) [15] allows the evaluation of the prognostic significance of molecular biomarkers in cancer samples in terms of survival outcomes in patients with breast, ovarian, lung, gastric, and other cancer types. We analyzed the prognostic value of *PSMDs* mRNA in ovarian cancer using hazard ratio (HR) and 95% confidence interval (CI) for survival outcomes. The prognostic value of high and low gene expression groups was evaluated using log-rank test. *P* < 0.05 was considered indicative of statistical significance.

### GeneMANIA analysis

GeneMANIA (http://www.genemania.org) [16] is used to generate a database of gene functions. On the basis of querying genes, GeneMANIA expands the list of genes with similar functions, indicating the relationship between genes and datasets. To draw the interactive functional association network, we used the database to construct *PSMDs* gene interaction network in the aspects of physical interaction, co-expression, prediction, co-localization, and genetic interaction, and evaluated the related functions.

### Function and pathway enrichment analysis

We obtained *PSMD8* co-expressed genes through the cBioPortal database. GO function and KEGG pathway enrichment analysis were applied using DAVID (https://david.ncifcrf.gov/) [17–20], which integrated biological data and analysis tools to provide a systematic synthesis Annotation information of biological functions. *P* < 0.05 was set as the cut-off criterion.

### Sample sources and clinical data

A total of 125 ovarian tissue paraffin specimens from inpatients who underwent surgical resection from 2008 to 2016 in the Department of Obstetrics and Gynecology, Shengjing Hospital Affiliated to China Medical University were selected. There were 80 cases of ovarian epithelial malignant tumor, 18 cases of ovarian epithelial borderline tumor, 16 cases of ovarian epithelial benign tumor, and 11 cases of normal ovarian tissue. There were no significant between-group differences with respect to age (*P* > 0.05). The pathological types of the malignant group included serous carcinomas (*n* = 58), mucinous carcinomas (*n* = 3), endometrioid carcinomas (*n* = 10), and clear cell carcinomas (*n* = 9). The malignant group was classified according to histology: 30 cases with high and medium differentiation and 50 cases with low differentiation. The surgical and pathological staging was performed according to the International Federation of Obstetrics and Gynecology (FIGO) criteria. In the malignant group, 66 patients underwent lymph node dissection, 41 patients had lymph node metastasis, 25 patients had no lymph node metastasis; the remaining patients did not undergo lymph node dissection. All the cases were primary, and no radiotherapy and chemotherapy were performed. Complete clinical data was available for all cases.

### Immunohistochemistry

The histopathological specimens used in the experiments were fixed in 10% formalin solution, paraffin-embedded, and serially sectioned at 5 µm. The paraffin sections were deparaffinised with xylene and re-hydrated with gradient alcohol solutions, and the antigens were recovered by heating. Subsequently, H_2_O_2_, goat serum blocking solution, and anti-PSMD8 antibody (1:100, ab246883, Abcam, Cambridge, UK) was added; the solutions were left to incubate overnight at 4 °C. The following day, the slices were incubated with horseradish peroxidase labelled goat anti-rabbit/mouse secondary antibodies and stained using 3,3-diaminobenzidine (UltraSensitive™ SP Mouse/Rabbit IHC Kit, Fuzhou Maixin Biotech Co. Ltd., Fuzhou, China). Nuclei were stained blue using hematoxylin. The sections were then dehydrated, cleared by xylene, and mounted.

Scoring method: Brownish-yellow, or brown colour of cytoplasm and cell membrane was considered as positive results. Brownish-yellow, brown, light yellow and no staining were scored as 3 points, 2 points, 1 point, and 0 points, respectively according to the coloring intensity. After observing the percentage of stained area in the whole section, the percentage of positive cells, > 75%, 51–75%, 26–50%, 5–25%, and < 5% were recorded as 4 points, 3 points, 2 points, 1 point, and 0 point, respectively. The final score was obtained by multiplying the two items: 0–2 points were recorded as negative expression(-), 3–4 points were recorded as weak positive expression( +), 5–8 points as moderately positive expression (+ +), and 9–12 points as strongly positive expression (+ + +). The sections were evaluated and scored independently by two pathologists who were blinded to the patient's information. The study was approved by the Ethics Committee of Shengjing Hospital Affiliated to China Medical University (2022PS411K). All methods were carried out in accordance with relevant guidelines and regulations.

### Cell culture

Ovarian cancer cell lines OVCAR3 and A2780 cell lines (Shanghai Institute of Biochemistry and Cell Biology, Chinese Academy of Sciences, Shanghai, China) were cultured in cell culture medium (Biological Industries, Beit-Haemek, Israel) containing 10% fetal bovine serum (Biological Industries, Beit-Haemek, Israel). When the cells had grown to 80–90% confluence, the culture medium was discarded, cells washed with PBS, and digested with trypsin, and stopped immediately when the cells were about to no longer adhere to the wall. The cells were centrifuged and resuspended, placed into a new culture vessel, and continued to culture.

#### Establishment of stably overexpressing PSMD8 cell line

Viral transfection was performed in OVCAR3 cell line and A2780 cell line using lentivirus-mediated *PSMD8* gene overexpression vector. First 500 µL of complete medium was added to the 24-well plate, followed by addition of *PSMD8* overexpressing lentivirus, and the corresponding volume of polybrene to facilitate transfection. The *PSMD8* stable high expression cell line OVCAR3-PSMD8-H and its control cell line OVCAR3-PSMD8-H-Mock, A2780-PSMD8-H and its control cell line A2780-PSMD8-H-Mock were constructed.

#### Establishment of transient low-expressing PSMD8 cell line

*PSMD8* siRNA was transfected in OVCAR3 and A2780 cell lines. The *PSMD8* siRNA working solution was prepared according to the instructions. On the day of transfection, the cells to be transfected were starved in a 6-well plate with serum-free medium. Serum-free medium, Lipo 3000, 7 µL *PSMD8* siRNA-1(5’ GCAUGUACGAGCAACUCAATT 3’ and 3’ UUGAGUUGCUCGUACAUGCTT 5’) or *PSMD8* siRNA-2 (5’ GACACUAUCAGGGAUGAGATT 3’ and 3’ UCUCAUCCCUGAUAGUGUCTT 5’) (GenePharma, Suzhou, China) was added to the cells to be transfected. After culturing the stained cells for 6–8 h, serum was added and the cells continued to culture for 48–72 h for cell function experiments. The PSMD8 low-expressing cell lines OVCAR3-PSMD8-L1, OVCAR3-PSMD8-L2 and their control cell lines OVCAR3-PSMD8-Mock, A2780-PSMD8-L1, A2780-PSMD8-L2 and their control cell lines A2780-PSMD8-Mock were constructed.

#### Scratch test

After observing the cells under the microscope to ascertain the ideal density, the pipette tip was used to make a cross scratch and pictures obtained. After culturing in serum-free medium for 24 h, the six-well plate was taken out, washed with PBS, and photographed to observe the healing of the scratches. The above experiment was repeated 3 times.

#### MTT experiment

Cells were seeded at a density of 2000 cells per well, and cultured at 37 °C. When the cells had adhered, the initial plate was counted as 0 h. 20 µL of sterile MTT working solution was added to each well, mixed well, and incubated at 37 °C for 4 h. Then, the medium was aspirated, 150 µL of DMSO blue-purple crystals were added, and the absorbance of each well was measured. Attention was paid to avoid light throughout the operation. The above experiment was repeated 3 times.

#### Invasion assay

The transwell chamber was placed in the culture plate. 1:7.5 diluted Matrigel (356,234, BD Biosciences, Franklin Lakes, NJ) and serum-free cell suspension (containing 4 × 10^4^ cells) were added to the upper chamber of the chamber, and serum-containing medium was added to the lower chamber. After culturing for 48–72 h, the cells in the lower chamber were taken out, fixed and stained. Residual cells left on plate were observed under a microscope. The above experiment was repeated 3 times.

#### Statistical analysis

SPSS 22.0 (IBM Corporation, Armonk, NY, USA) software was used for data analysis. Chi-squared test and Fisher exact test were used for enumeration data, and *t* test was used for measurement data. Kaplan–Meier method was used to generate survival curves and between-group differences were assessed using log-rank test. Cox regression models were used to analyze the relationship between PSMD8 expression and clinicopathological parameters. *P* values < 0.05 were considered indicative of statistical significance.

## Results

### Different expression of PSMD family in ovarian tissue

**Fig. 1:**
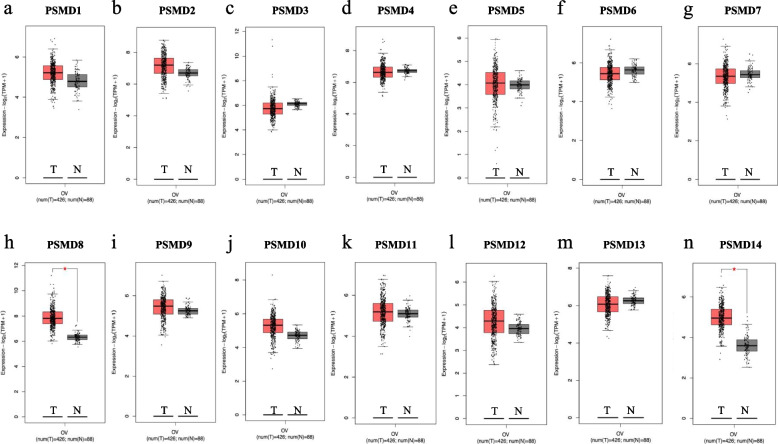
mRNA expression of *PSMDs* in ovarian cancer tissue and normal ovarian tissue (GEPIA database). **a**-**n** mRNA expression of *PSMD*1-14 members in ovarian cancer tissue and normal ovarian tissue. T represented tumor tissuse and N represented normal tissues

Differences in mRNA expression of *PSMDs* in ovarian cancer and normal ovarian tissues were analyzed using GEPIA database (Fig. 1). The mRNA expression levels of *PSMD8* and *PSMD14* in ovarian cancer tissues were significantly higher than those in normal ovarian tissues (Fig. 1h, n).

**Fig. 2:**
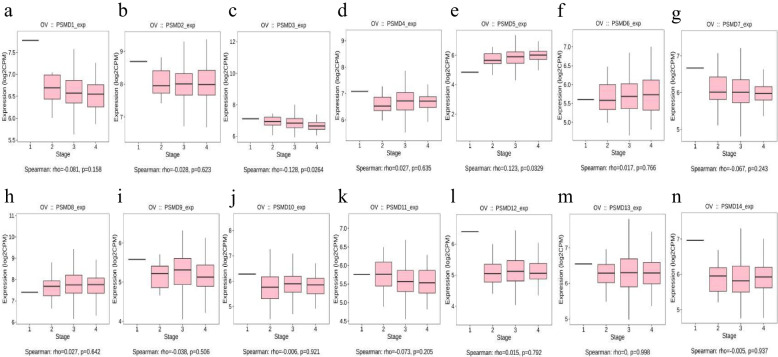
Correlation analysis between the expression of *PSMDs* and clinical stage in ovarian serous adenocarcinoma (TISIBD database). **a**-**n** The expression of each member of *PSMD1-14* in ovarian serous adenocarcinoma was correlated with clinical stage

The relationship between *PSMDs* mRNA expression level and clinical stage in ovarian serous adenocarcinoma was further analyzed using the TISIBD database (Fig. 2). The results showed that *PSMD3* expression significantly decreased with increase in FIGO stage (Fig. 2c, Spearman: rho = 0.128, *P* = 0.0264), while *PSMD5* expression increased significantly with increase in FIGO stage (Fig. 2e, Spearman: rho = 0.123, *P* = 0.0329).

### Gene variation in PSMDs in ovarian cancer

**Fig. 3:**
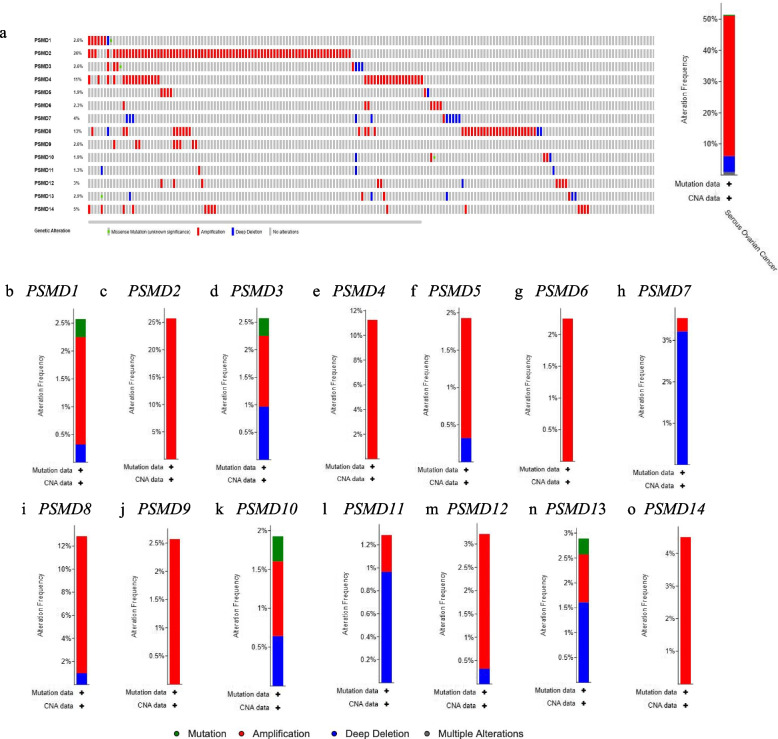
Gene variation analysis of *PSMDs* in ovarian cancer (cBioPortal database). **a** Overview of *PSMDs* gene variation analysis. **b**-**o** Gene variation analysis of *PSMD1-14* members in different studies

Genetic variations of *PSMDs* in 617 cases retrieved from three studies (617 cases from TCGA, Firehose legacy) were analyzed using the cBioPortal database (Fig. 3). We found varying degrees of genetic variation among the 14 *PSMD* family members, among which *PSMD2* displayed the highest incidence rate ( 25.72% in TCGA) of genetic variations (the incidence rates of amplification) (Fig. 3b), followed by *PSMD8* whose incidence rates of amplification and deep deletion were 11.90% and 0.96%, respectively (in TCGA) and *PSMD4* whose incidence rate of amplification was 11.25%. (Fig. 3e, i).

### PSMD8 showed the best prognostic value in patients with ovarian serous carcinomas

**Fig. 4:**
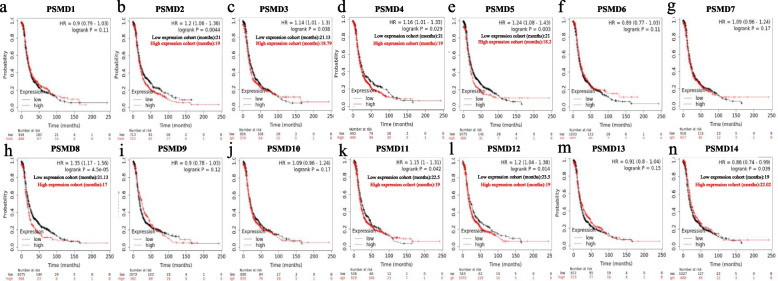
Prognostic value of *PSMDs* expression levels in ovarian cancer (PFS in Kaplan–Meier Plotter). **a**-**n** Prognostic significance of individual *PSMD1-14* members in ovarian tumor

The correlation between *PSMDs* mRNA expression levels and PFS in ovarian cancer patients was analyzed using the Kaplan–Meier Plotter database (Fig. 4). Among them, *PSMD2, PSMD3, PSMD4, PSMD5, PSMD8, PSMD11, PSMD12,* and *PSMD14* mRNA expression levels were associated with prognosis. These findings indicate the prognostic significance of *PSMDs* in the context of ovarian cancer.

**Fig. 5:**
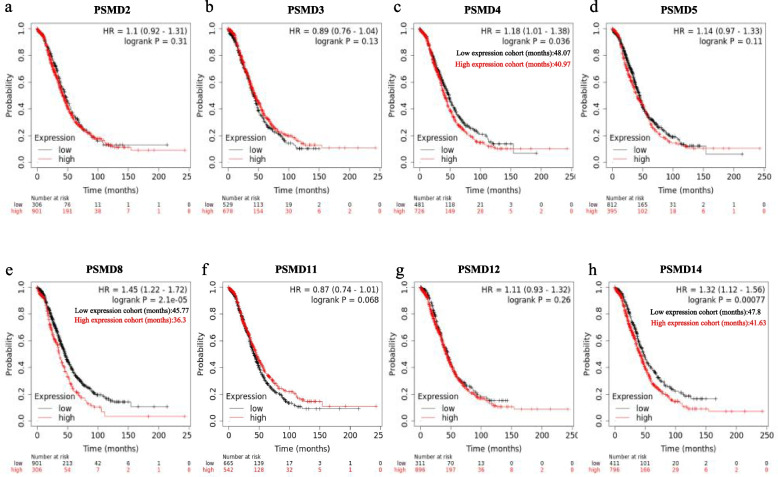
Survival analysis of *PSMDs* in ovarian serous carcinomas (OS in Kaplan–Meier Plotter)

**Fig. 6:**
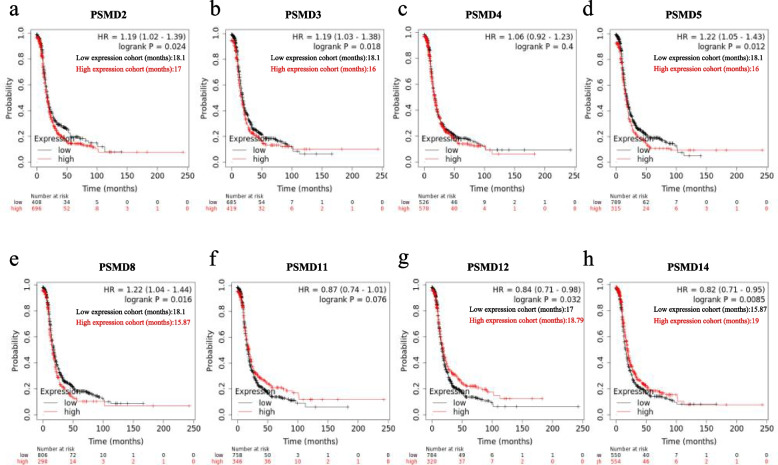
Survival analysis of *PSMDs* in ovarian serous carcinomas (PFS in Kaplan–Meier Plotter)

Ovarian serous carcinomas are the most common pathological type of ovarian carcinomas. Therefore, we assessed the correlation of the expression of the above-mentioned 8 genes in ovarian serous carcinomas with OS and PFS (Figs. 5 and 6). Among them, up-regulation of *PSMD4, PSMD8,* and *PSMD14* mRNA expression was significantly associated with poor OS in patients with ovarian serous carcinomas (Fig. 5c,e,h). Up-regulation of *PSMD2, PSMD3, PSMD5,* and *PSMD8* mRNA expression was significantly associated with poor PFS in patients with ovarian serous carcinomas (Fig. 6a, HR = 1.19, 95% CI: 1.02–1.39, *P* = 0.024; Fig. 6b, HR = 1.19, 95% CI: 1.03–1.38, *P* = 0.018; Fig. 6d, HR = 1.12, 95% CI: 1.05–1.34, *P* = 0.012; Fig. 6e, HR = 1.22, 95% CI: 1.04–1.44, *P* = 0.016), while down-regulation of *PSMD12* and *PSMD14* mRNA expression was associated with poor PFS (Fig. 6g, HR = 0.84, 95% CI: 0.71–0.98, *P* = 0.032; Fig. 6h, HR = 0.82, 95% CI: 0.71–0.95, *P* = 0.0085). On comprehensive comparison, up-regulation of *PSMD8* mRNA expression was significantly associated with poor OS and PFS in patients with ovarian serous carcinomas.

**Fig. 7:**
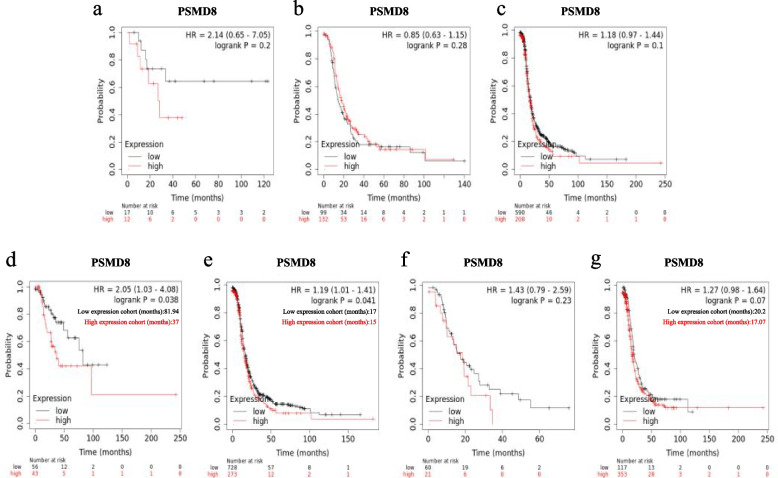
Survival analysis of *PSMD8* in ovarian serous carcinomas (PFS in Kaplan–Meier Plotter). **a**-**c** Prognostic significance of *PSMD8* in ovarian serous carcinoma with different grade. **d**-**e** Prognostic significance of *PSMD8* in ovarian serous carcinoma with different FIGO stage. **f** Prognostic significance of *PSMD8* in ovarian serous carcinoma without TP53 mutation. **g** Prognostic significance of *PSMD8* in ovarian serous carcinoma with TP53 mutation

*PSMD8,* which showed the greatest prognostic significance in patients with ovarian serous carcinomas, was selected for correlation analysis. We separately assessed the correlation between *PSMD8* mRNA expression and PFS at different degrees of differentiation, FIGO stages, and TP53 mutation status (Fig. 7). There was no significant correlation between the up-regulation of *PSMD8* mRNA expression and poor PFS in patients with moderately- and poorly-differentiated carcinomas (Fig. 7a-c); in patients with FIGO stage III-IV, the up-regulation of *PSMD8* mRNA expression indicated poor PFS (Fig. 7d-e). Compared with wild type, *PSMD8* mRNA upregulation in TP53 mutant patients was associated with significantly poor PFS (Fig. 7f). These findings indicated that *PSMD8* can better reflect the prognosis of patients with ovarian serous carcinomas, and in patients with advanced FIGO stage and TP53 mutation, *PSMD8* showed a more significant correlation with prognosis.

### PSMDs gene interaction network construction and enrichment analysis

**Fig. 8:**
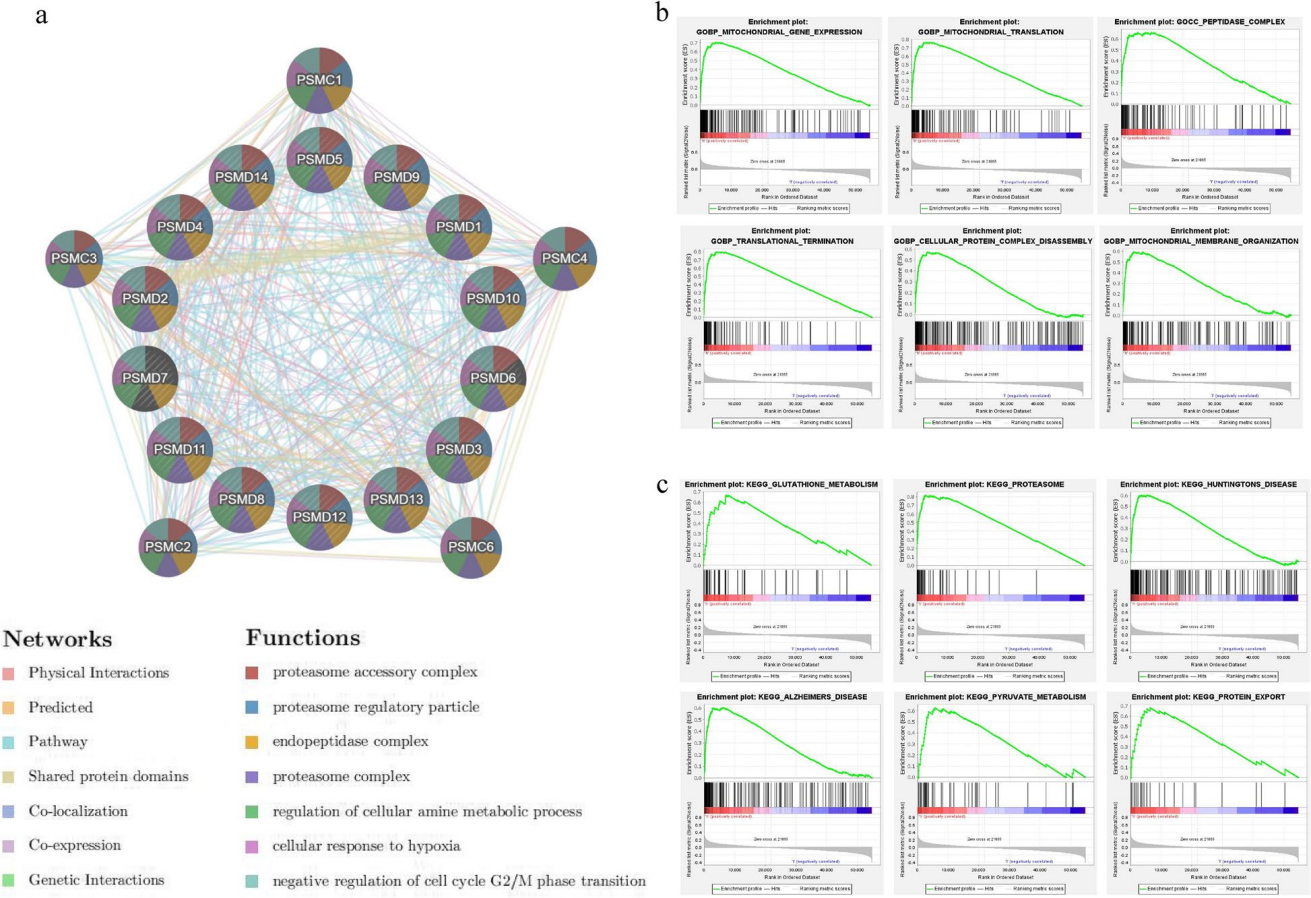
Spearman's correlation analysis, GO function and KEGG pathway enrichment analysis. **a** Construction of the gene interaction network of each member of *PSMDs* (each node represents a gene, the size of the node represents the strength of the interaction, and the lines between nodes represent different ways of interaction between genes). **b**, **c** The function and pathway enrichment analysis of *PSMD8* co-expression genes

The gene interaction network map of the 14 genes of *PSMDs* was constructed using the GeneMANIA database, and the correlations were analyzed (Fig. 8a). The 14 nodes in the middle are members of *PSMDs*, and the surrounding 20 nodes are the 20 genes most related to the family in terms of physical interaction, interaction, co-localization, prediction, inheritance, and co-expression. The five most related genes are *PSMC1, PSMC4, PSMC6, PSMC2*, and *PSMC3*, which are members of the *PSMCs* family.

The function and pathway enrichment analysis of *PSMD8* co-expressed genes was carried out using the database. Among the signal pathways with strong correlation of *PSMDs* co-expressed genes, glutathione metabolism, pyruvate metabolism, DNA replication, arginine and proline metabolism were related to the occurrence and development of tumors. Functional analysis showed that *PSMD8* co-expressed genes were mainly enriched in the following biological processes, including mitochondrial gene expression, mitochondrial translation, peptidase complex, translation termination, cellular protein complex disassembly, and mitochondrial membrane organization (Fig. 8b, c).

### PSMD8 is involved in the regulation of immune molecules

**Fig. 9:**
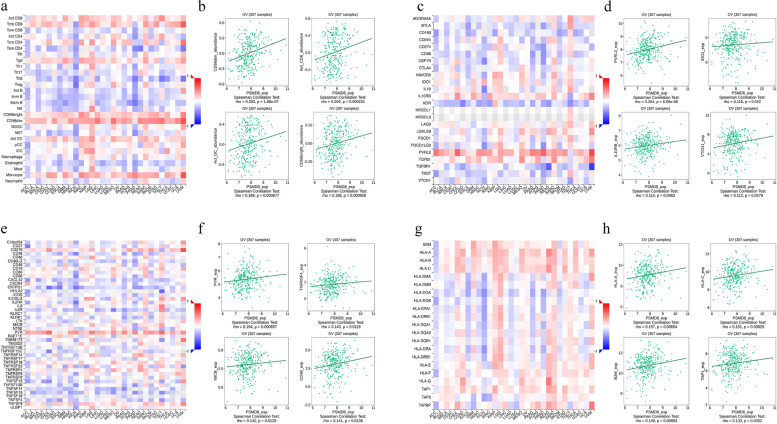
Spearman correlation of *PSMD8* with lymphocytes and immunomodulators (TISIDB database). **a** Relations between abundance of tumor-infiltrating lymphocytes (TILs) and expression of *PSMD8.* **b** Four with the highest Spearman correlation with *PSMD8.* **c** Relations between abundance of immunoinhibitor and expression of *PSMD8*. **d** Four immunosuppressants with the highest Spearman correlation with *PSMD8*. **e** Relations between abundance of immunostimulator and expression of *PSMD8*. **f** Four immunostimulants with the highest Spearman correlation with *PSMD8*. **g** Relations between abundance of MHC molecule and expression of *PSMD8*. **h** Four MHC molecules with the highest correlation with *PSMD8* Spearman

Spearman's correlation analysis was performed to assess the correlation of *PSMD8* expression with lymphocyte subsets and immunomodulators using the TISIDB database. Figure 9a and b showed the correlation between *PSMD8* expression and tumor-infiltrating lymphocytes (TILs). The lymphocyte subsets displaying the greatest correlations included *CD56dim* (Spearman: *ρ* = 0.293, *P* = 1.88e − 07), *Act_CD8* (Spearman: *ρ* = 0.209, *P* = 0.000231), *Act_DC* (Spearman: *ρ* = 0.189, *P* = 0.000877), and *CD56bright* (Spearman: *ρ* = 0.188, *P* = 0.000928). Immunomodulators were further classified into immunoinhibitors, immunostimulators, and major histocompatibility complex (MHC) molecules. Figure 9c and d showed the correlation of *PSMD8* expression levels with immunoinhibitors. The immunoinhibitors displaying the greatest correlations included *PVDL2* (Spearman: *ρ* = 0.254, *P* = 6.99e − 06), *IDO1* (Spearman: *ρ* = 0.116, *P* = 0.042), *IL10RB* (Spearman: *ρ* = 0.114, *P* = 0.0463), and *VTCN1* (Spearman: *ρ* = 0.113, *P* = 0.0479). Figure 9e and f showed the correlation between immunostimulators and *PSMD8*; the immunostimulators displaying the strongest correlation included *PVR* (Spearman:*ρ* = 0.194, *P* = 0.000657), *TNFRSF4* (Spearman:*ρ* = 0.143, *P* = 0.0119), *MICB* (Spearman:*ρ* = 0.142, *P* = 0.0129), and *CD48* (Spearman: *ρ* = 0.141, *P* = 0.0136). Figure 9g and h showed correlations between *PSMD8* expression and MHC molecules. The MHC molecules displaying the strongest correlation included *HLA-A* (Spearman:*ρ* = 0.157, *P* = 0.00604), *HLA-C* (Spearman:*ρ* = 0.151, *P* = 0.00829), *B2M* (Spearman:*ρ* = 0.149, *P* = 0.00893), and *TAP1* (Spearman:*ρ* = 0.133, *P* = 0.0202).

### PSMD8 is highly expressed in different kinds of cancer tissues

**Fig. 10:**
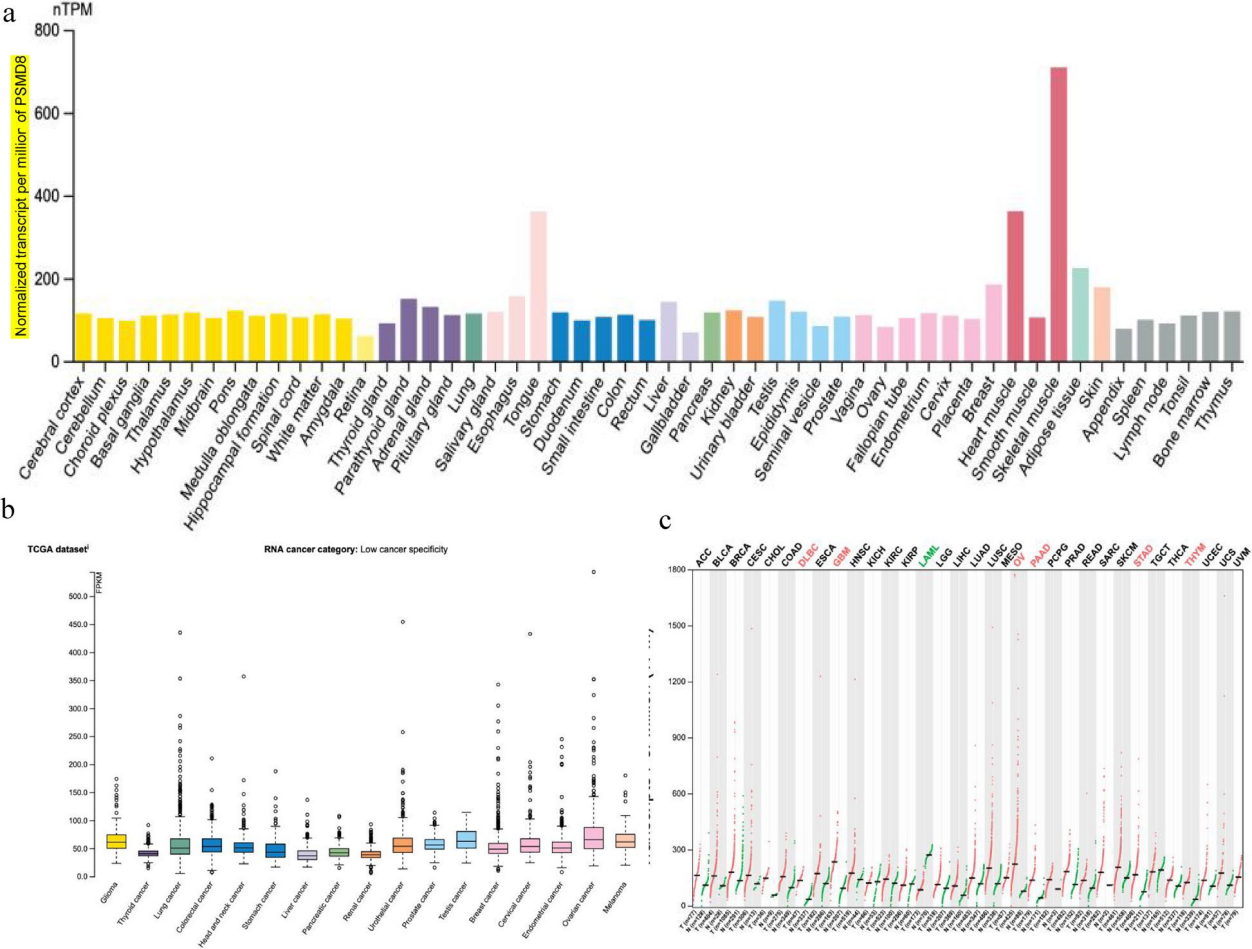
*PSMD8* is highly expressed in different kinds of cancer tissues. **a** *PSMD8* mRNA expression in various normal tissues and various cancer tissues (human protein atlas website). **b** *PSMD8* expression in different types of cancer tissues (TCGA). **c** *PSMD8* gene expression profile across all tumor samples and paired normal tissues. Each dots represent expression of samples (GEPIA)

The mRNA expression of *PSMD8* in normal tissues and cancer tissues was analyzed using the human protein atlas website, and the results showed greater expression of *PSMD8* in normal skeletal muscle, cardiac muscle, and tongue muscle (Fig. 10a). TCGA database showed that among malignant tumors, *PSMD8* was more frequently expressed in ovarian cancer, testicular cancer, glioma, and melanoma (Fig. 10b). GEPIA website data analysis showed that *PSMD8* is highly expressed in ovarian cancer, pancreatic cancer, gastric cancer, thymic cancer, glioblastoma multiforme, and diffuse large B-cell lymphoma, while it is lowly expressed in acute myeloid leukemia. In conclusion, *PSMD8* has a higher abnormal expression in ovarian cancer (Fig. 10c).

### Immunohistochemistry confirmed the high expression of PSMD8 in ovarian cancer tissue

**Fig. 11:**
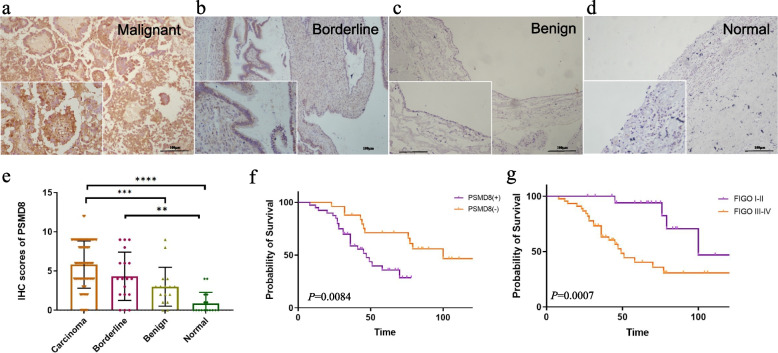
PSMD8 expression in clinical specimens. **a**-**d** The expression of PSMD8 in the same position in malignant, borderline, benign, and normal tissues. **e** PSMD8 scores in different ovarian tissues. **f** The influence of PSMD8 expression on the survival of ovarian cancer patients. **g** The influence of FIGO stage on the survival of ovarian cancer patients

PSMD8 is mainly located in the cytoplasm and its expression is indicated by brown staining (Fig. 11a-d). The positive rate in the malignant group was 96.19%, and the strong positive rate was 70.48%. In the borderline group, the positive rate was 41.67%, and the strong positive rate was 16.67%. In the benign group, the positive rate was 16.67% and the strong positive rate was 11.11%. In normal ovarian tissue, the positive rate was 6.25%, and the strong positive rate was 0.00%. The positive expression rate and strong positive rate of PSMD8 in the malignant group were significantly higher than that in borderline group, benign group, and normal group (*P* < 0.05 for all). The positive expression rate of PSMD8 in the borderline group was greater than that in the benign group and normal group (*P* < 0.05). The expression rate of PSMD8 in the benign group was higher than that in the normal group, but the difference was not statistically significant (*P* > 0.05) (Table 1, Fig. 11e).

**Table 1:** Expression of PSMD8 in different ovarian tissues

Groups	Cases	Low		High		Positive Rate (%)	High expression Rate (%)
		**-**	** + **	** + + **	** + + + **		
Malignant	80	13	20	24	23	83.75%	58.75%
Borderline	18	5	5	3	4	66.67%	38.89%
Benign	16	7	7	1	1	56.25%	12.50%
Normal	11	9	2	0	0	18.18%	0.00%

### Relationship between PSMD8 expression and clinicopathological parameters of ovarian cancer

In order to compare the clinicopathological parameters and the expression of PSMD8 in ovarian tissue, we collected the pathological information of 80 patients with primary ovarian epithelial malignant carcinomas. The strong positive expression rate was significantly higher than that in FIGO I ~ II group (80.33% and 56.82%, *P* < 0.01), while there was no significant difference in other items (Table 2).

**Table 2:** Relationships between the expression of PSMD8 and clinicopathological parameters of 80 ovarian cancer patients

Groups	Cases	PSMD8			
		Positive rate(%)	*P-*value	High expression rate(%)	*P-*value
**Age at diagnosis**					
< 55	42	37/42(88.10%)	*0.366*	27/42(64.29%)	*0.365*
≥ 55	38	30/38(78.95%)		20/38(72.63%)	
**Pathological type**					
Serous	58	50/58(86.21%)	*All P* > 0.05*	33/58(56.90%)	*All* *P* > 0.05**
Mucious	3	2/3(66.67%)		1/3(33.33%)	
Endometrioid	10	7/10(70.00%)		6/10(60.00%)	
Clear cell carcinoma	9	8/9(88.89%)		7/9(77.78%)	
**FIGO stage**					
I-II	26	15/26(57.69%)	*0.0001*	7/26(26.92%)	*0.0001*
III-IV	54	52/54(96.30%)		40/54(74.07%)	
**Differentiation**					
Well-moderate	30	23/30(76.67%)	*0.183*	14/30(46.67%)	*0.089*
Poor	50	44/50(88.00%)		33/50(66.00%)	
**Lymphatic metastasis**					
No	40	32/40(80.00%)	*0.403*	18/40(47.50%)	0.070
Yes	25	22/25(92.00%)		17/25(68.00%)	
Unknown^a^	15	13/15(86.67%)		12/15(80.0%)	

^*^Serous vs mucious 0.351, Serous vs endometrioid 0.199, serous vs clear cell carcinoma 0.826, mucious vs endometrioid 0.913, mucious vs clear cell carcinoma vs 0.371,endometrioid vs clear cell carcinoma 0.313

^**^Serous vs mucious 0.423, Serous vs endometrioid 0.855, serous vs clear cell carcinoma 0.235, mucious vs endometrioid 0.417, mucious vs clear cell carcinoma vs 0.157, endometrioid vs clear cell carcinoma 0.405

^a^Patients without lymphadenectomy

### Prognostic significance of PSMD8 expression in ovarian cancer patients

On follow-up of patients, it was found that 13 deaths occurred in the PSMD8 low expression group (*n* = 30), as compared to 23 deaths in the PSMD8 high expression group (*n* = 50). Kaplan–Meier survival analysis showed that the survival rate of patients in the PSMD8 high expression group was significantly shorter than that in the PSMD8 low expression group, and the survival rate of patients in FIGO stage III ~ IV was significantly lower than that in FIGO stage I ~ II (*P* < 0.05) (Fig. 11f,g).

We performed univariate and multivariate Cox regression analysis to assess the influence of PSMD8 expression, age, pathological type, degree of differentiation, FIGO stage, and lymph node metastasis on postoperative survival time of patients. The results indicated that PSMD8 expression and FIGO stage were prognostic risk factors for epithelial ovarian malignancies (Table 3).

**Table 3:** Univariate and Multivariate Cox Analysis of Different Clinicopathological Parameters with Ovarian Cancer

Variable	Categories	Univariate analysis			Multivariate analysis		
		HR	95% CI of HR	*P*	HR	95% CI of HR	*P*
Age at diagnosis	< 55	0.875	0.450 – 1.701	0.694			
	≥ 55						
FIGO stage	I-II	2.562	1.153 – 5.695	0.021*	1.992	0.863 –4.597	0.106
	III-IV						
Differentiation	Well-moderate	1.099	0.555—2.176	0.786			
	Poor						
Lymphnode metastasis	No	1.864	0.858 – 4.051	0.116			
	Yes						
PSMD8	Low	2.645	1.106 – 6.325	0.029*	2.424	1.034 – 5.681	0.042*
	High						

#### PSMD8 promotes the invasion, migration and proliferation of ovarian cancer cells

**Fig. 12:**
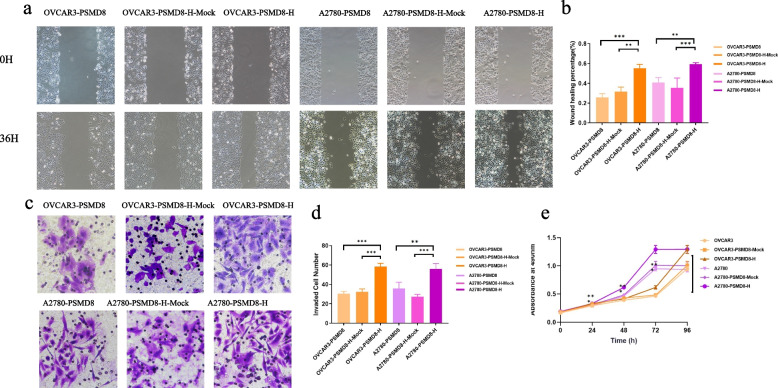
High expression of PSMD8 enhances cell invasion, migration and MTT in ovarian cancer. **a** **b** Effects of high PSMD8 expression on the migration of ovarian cancer in OVCAR3 and A2780 cells. **c**, **d** Effects of high PSMD8 expression on the invasion of ovarian cancer in OVCAR3 and A2780 cells. **e** Effects of high PSMD8 expression on the proliferation abilities of ovarian cancer in OVCAR3 and A2780 cells

**Fig. 13:**
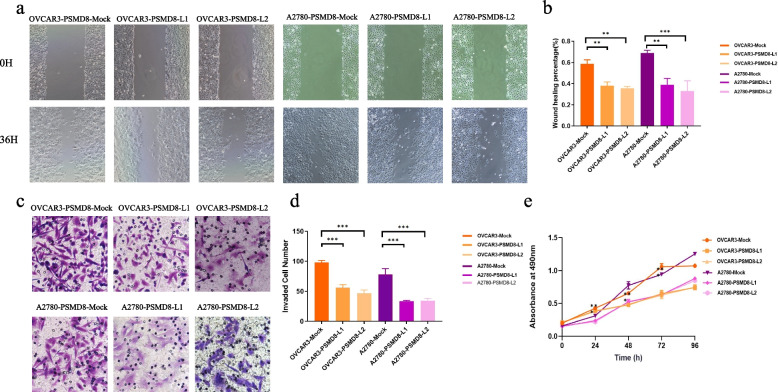
Low expression of PSMD8 enhances cell invasion, migration and MTT in ovarian cancer. **a**, **b** Effects of low PSMD8 expression on the migration of ovarian cancer in OVCAR3 and A2780 cells. **c**, **d** Effects of low PSMD8 expression on the invasion of ovarian cancer in OVCAR3 and A2780 cells. **e** Effects of low PSMD8 expression on the proliferation abilities of ovarian cancer in OVCAR3 and A2780 cells

After differential expression of PSMD8, the effects on the invasion, migration, and proliferation of ovarian cancer cells were detected by transwell assay, cell scratch assay, and MTT assay. The results showed that: OVCAR3-PSMD8-H and A2780-PSMD8-H cells had significantly stronger invasion, migration, and proliferation abilities than the control group OVCAR3-PSMD8-MOCK, OVCAR3 and A2780-PSMD8-MOCK, A2780. The ability of OVCAR3-PSMD8-L1/L2 and A2780-PSMD8-L1/L2 cells were significantly weaker than that of the control group OVCAR3-PSMD8-MOCK and A2780-PSMD8-MOCK in invasion, migration, proliferation (*P* < 0.05 for both) (Figs. 12 and 13). The results indicated that PSMD8 promoted the invasion, migration, and proliferation ability of ovarian cancer cells.

## Discussion

Ubiquitination is an important post-translational modification that controls substrate degradation and can be reversed by deubiquitinating enzymes (DUBs) [21]. The ubiquitin–proteasome system (UPS) is responsible for the degradation of 80% of intracellular proteins in eukaryotic cells [22]. The UPS is involved in a wide range of biological functions, such as cell growth, cell cycle progression, DNA transcription, damage, repair, and signal transduction [23, 24]. Therefore, dysfunction of the UPS or its components can lead to severe disease [25–29].

The proteasome is a multi-subunit complex consisting of a 19S regulatory granule and a 20S core granule, which mainly functions to degrade ubiquitin-tagged proteins [30]. Among them, the 19S regulatory particles are divided into two parts: the base and the lid, which are connected to the outer surface of the 20S core particles and play the role of recognizing ubiquitinated protein substrates, removing ubiquitin linkages, unfolding proteins, and transporting proteins into the 20S core particles. The 20S core particle is responsible for protein degradation [31]. *PSMD*s encodes a family of subunits of the 26S proteasome, which is a non-ATPase subunit in the proteasome structure. It has 14 members in total that participate in the formation of the 19S regulatory complex and perform the functions of catalyzing the unfolding and transport of substrate proteins. *PSMDs* play an important role in a variety of cancers, and abnormal gene expression is often associated with tumor-regulating oncogenes and tumor suppressor genes [9–11, 32–34]. Thus, these are potential diagnostic and prognostic biomarkers as well as therapeutic targets.

In a study, loss of PSMD1 was found to inhibit the proliferation of breast cancer cells and induce cell cycle arrest by inhibiting the degradation of p53. The upregulation of PSMD1 gene was mainly accompanied by increase of tamoxifen resistance in BRCA cells [35]. High PSMD1 expression was shown to be significantly associated with disease-free survival (DFS) and overall survival (OS) of gastric cancer patients [36]. PSMD2 and PSMD7 were shown to regulate breast cancer cell proliferation and cell cycle progression by regulating the proteasomal degradation of p21 and p27 [37]. PSMD3 regulates breast cancer by stabilizing HER2 degradation [38]. PSMD4 was shown to affect esophageal cancer by inhibiting endoplasmic reticulum stress and degree of cellular malignancy [39]. Antioxidant response element-bound nuclear Nrf2 (nNrf2) promotes chemoresistance in colorectal cancer through the EMT pathway via the NF-κB/AKT/β-catenin/ZEB1 cascade by inducing PSMD4 expression [40]. PSMD4 copy number amplification was associated with sensitivity to Poly (ADP-ribose) polymerase (PARP) inhibitors (PARPi), and it might be a better predictor of PARPi sensitivity than BRCA1/2 mutations [41]. Inactivation of PSMD5 was shown to promote colorectal tumor progression [42], TNF-α increases PSMD5 expression through NFκB. Excess PSMD5 directly inhibited the assembly and activity of the 26S proteasome, and TNF-α enhanced the interaction of PSMD5 with PSMC2. In another study, the expression of PSMD6, PSMD9, PSMD11, and PSMD14 was significantly associated with a decreased chance of survival in patients with pancreatic ductal adenocarcinoma [43]. PSMD7 was considered an oncogene in prostate cancer, esophageal squamous cell carcinoma (ESCC), and breast cancer [44, 45]. PSMD7 knockout was shown to induce cell cycle arrest in G0/G1 phase, leading to cell senescence and apoptosis or inhibit lung cancer progression by modulating the p53 pathway [45].

PSMD8, a deubiquitinating enzyme, is a member of the JAMM (JAB1/MPN/Mov34) domain family, and studies have shown that PSMD8 interacts with the sperm adhesin AQN1 to limit polyfertilization [46]. It was highly expressed in invasive bladder cancer and breast cancer [47, 48]. High expression of PSMD9 was associated with post-radiotherapy recurrence in cervical and breast cancer [49], and endogenous PSMD10 interacted with GRP78 to regulate endoplasmic reticulum stress, which might provide a therapeutic target for homocysteine-induced liver injury [50]. miR-3619-5p inhibited tumor growth in vivo by inducing the phosphorylation of activator of transcription 3 (STAT3) and retinoblastoma protein (Rb1), thereby targeting PSMD10 to inhibit cell proliferation and induce G1 arrest [51]. PSMD11 and PSMD12 have been extensively studied in the nervous system. The expression of PSMD11 was down-regulated in the hippocampus of epileptic mice, and the lncRNA Peg13 was shown to up-regulate PSMD11 in a miR-490-3p-dependent manner, thereby inactivating the Wnt/β-catenin pathway and relieving epilepsy progression in mice [52]. Bi et al. [53] reported that silencing the *PSMD13* gene has the potential to treat neuroinflammatory diseases by regulating the activation of microglia and the production of inflammatory mediators. PSMD12 enhanced the proliferation and invasion of glioma cells through Akt signaling-mediated Nrf2 expression [54], and PSMD12 was considered to be a key regulator of glioma development and progression. PSMD14 had been shown to play an oncogenic role in the context of ovarian, prostate, hepatocellular, lung adenocarcinoma, and colorectal cancers [55–59]. PSMD14 overcame drug resistance in head and neck squamous cell carcinoma by inhibiting E2F1 ubiquitination and degradation, improving Akt pathway activation and SOX2 transcription [60].

In this study, analysis of mRNA expression data of PSMD8 in various normal tissues and various cancer tissues showed that PSMD8 was more expressed in normal skeletal muscle, cardiac muscle, and tongue muscle; among malignant tumors, PSMD8 was expressed in ovarian cancer, testicular cancer, glioma, and melanoma. On analyzing the relationship of *PSMD* family and ovarian cancer, the expression levels of *PSMD8* and *PSMD14* mRNA in ovarian cancer were found to be significantly higher than those in normal ovarian tissue. Prognostic analysis found that patients with high mRNA expression of *PSMD2, PSMD3, PSMD4, PSMD5, PSMD8, PSMD11, PSMD12,* and *PSMD14* have poor prognosis; among these, *PSMD8* showed the best prognostic value in patients with serous ovarian cancer. On analyzing the relationship of *PSMD8* expression with different stages, differentiation, and TP53 mutation status, *PSMD8* mRNA expression was found to be up-regulated in FIGO stage III-IV and TP53 mutant patients, and the PFS was worse. Furthermore, immunohistochemical experiments demonstrated that PSMD8 was mainly expressed in the cytoplasm, and was highly expressed in ovarian epithelial malignant tumor tissues, and the expression level showed a correlation with FIGO stage. Patients with high PSMD8 expression and advanced FIGO stage showed a poor prognosis. Therefore, PSMD8 had the strongest correlation with the prognosis of ovarian cancer patients, and was closely related to the occurrence and development of ovarian cancer. Through the analysis of spearman correlation of *PSMD8* with lymphocytes and immunomodulators of TISIDB database, the patients with *CD56dim, Act_CD8, Act_DC* and *CD56bright* showed high expression of PSMD8, indicating that patients with high expression of PSMD8 may have poor effect on immunotherapy. Due to the heterogeneity of tumors, studies have shown that TILs therapy had a good therapeutic effect, while not all tumors responded well which need to be validated in clinical trials. Furthermore, the immunoinhibitors including *PVDL2, IDO1*, *IL10RB* and *VTCN1,* immunostimulators including *PVR, TNFRSF4*, *MICB* and *CD48*; MHC molecules including *HLA-A*, *HLA-C*, *B2M*, and *TAP1* indicated the relationship between immune-related molecules and *PSMD8.* MTT, cell scratch assay, and transwell assay confirmed that PSMD8 overexpression could enhance malignant biological behaviors such as proliferation, migration, and invasion of ovarian cancer cells, indicating that PSMD8 could be used as a potential marker for early diagnosis, disease progression, and prognostic assessment in patients with ovarian cancer. Moreover, it was also a potential therapeutic target. The function and enrichment analysis of *PSMD*8 genes through the database showed that these genes are mainly involved in energy metabolism, such as glucose metabolism, mitochondrial energy metabolism, DNA replication, protein synthesis and other biological processes. Cancer-related signaling pathways affected the occurrence and development of ovarian cancer.

Based on the above studies, we identified the important role of the *PSMD* family, especially PSMD8, in the occurrence and development of ovarian cancer. In vitro experiments as well as analysis of the relationship of PSMD8 expression in ovarian cancer with the clinicopathological parameters and survival outcomes showed that PSMD8 overexpression can enhance malignant biological behavior of ovarian cancer. Our findings suggest that PSMD8 is a potential biomarker for early diagnosis, disease progression, and prognostic assessment of patients with ovarian cancer patients. In addition, it is also a potential therapeutic target. However, due to differences in database backgrounds, limited sample size, and lack of relevant experimental foundations, further experiments are required for more in-depth characterization of the specific roles and related mechanisms of PSMDs family and PSMD8 in ovarian cancer.

## Supplementary Information

**Additional file 1.**

## Data Availability

All data generated or analysed during this study are included in this published article (the websites provided) and its supplementary information files.
