# Supplementary material for: PSMD8 can serve as potential biomarker and therapeutic target of the PSMD family in ovarian cancer: based on bioinformatics analysis and in vitro validation
Source: BMC Cancer. 2023 Jun 22;23:573. doi: 10.1186/s12885-023-11017-8 (PMC10286491; doi:10.1186/s12885-023-11017-8)
Supplement: Supplementary file 1 — Additional file 1. [file 12885_2023_11017_MOESM1_ESM.pdf]

HIC-1

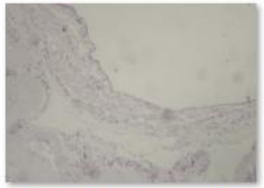

BENIGN10-LX-PSMD8.tif

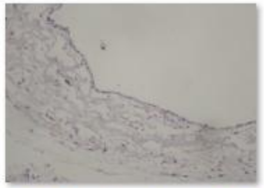

BENIGN20-LX-PSMD8.tif

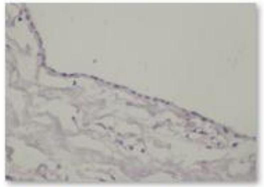

BENIGN-40-LX-PSMD8.tif

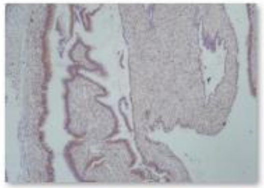

BORDER10-PSMD8.tif

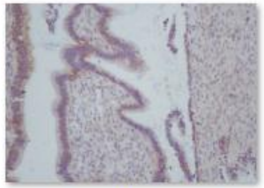

BORDER20-LX-PSMD8.tif

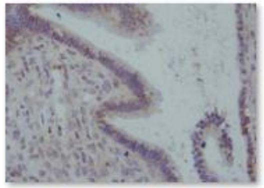

BORDER40-LX-PSMD8.tif

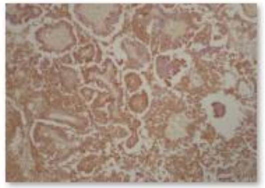

CANCER(10)1-10-LX-PSMD8.tif

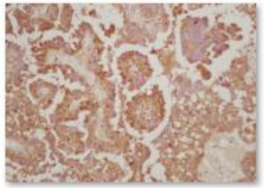

CANCER(20)1-20-LX-PSMD8.tif

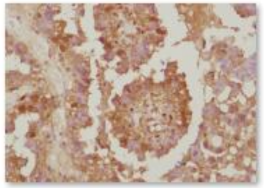

CANCER(40)1-40-LX-PSMD8.tif

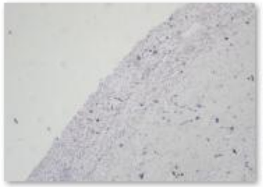

NORMAL10-LX-PSMD8.tif

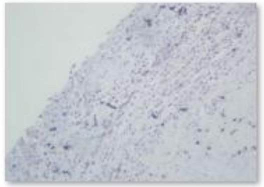

NORMAL20-LX-PSMD8.tif

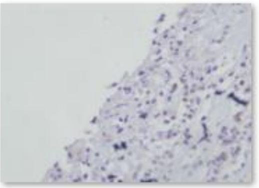

NORMAL40-LX-PSMD8.tif

HIC-2

NC	COLOR（0-3	AREA（0-4）	SCOI	Gro	FIG	FIG	Gra	Lymphnc	ag	Ag	Survival t	Dea	Tim		
[illegible]	3	3	9	2	IIIc	2	2	2	54	1	death：14	1	14		
[illegible]	3	3	9	2	IV	2	2	2	51	1	death：12	1	12		
[illegible]	3	2	6	2	III c	2	2	1	63	2	death：31	1	31		
[illegible]	2	4	8	2	IIIc	2	2	2	62	2	death：36	1	36		
[illegible]	0	4	0	2	IIIc	2	1	2	62	2	death：51	1	51		
[illegible]	3	3	9	2	IIIc	2	1	2	48	1	death；49	1	49		
[illegible]	2	3	6	2	IIIc	2	1	1	63	2	death：28	1	28		
[illegible]	2	4	8	2	IIIC	2	2	1	63	2	24	1	24		
[illegible]	2	4	8	2	IIIC	2	2	2	57	2	27	1	27		
[illegible]	3	3	9	2	IIIc	2	2	2	71	2	death：36	1	36		
[illegible]	3	3	9	2	IIIc	2	1		45	1	death：28	1	28		
[illegible]	3	3	9	2	IIIc	2	2		77	2	death：36	1	36		
[illegible]	3	3	9	2	IIIc	2	1	1	52	1	death：58	1	58		
[illegible]	3	3	9	2	IIIc	2	1		64	2	death：45	1	45		
[illegible]	3	3	9	2	IIIc	2	1		78	2	death：20	1	20		
[illegible]	3	3	9	2	IIIC	2	2	2	28	1	26	1	26		
[illegible]	2	3	6	2	IIIc	2	2	1	74	2	death：31	1	31		
[illegible]	3	3	9	2	III	2	1		53	1	death：41	1	41		
[illegible]	2	4	8	2	IIIc	2	2	1	71	2	death；70	1	70		
[illegible]	3	3	9	2	IIIc	2	2	2	43	1	death：8	1	8		
[illegible]	3	3	9	2	IIIc	2	2	2	42	1	death：47	1	47		
[illegible]	2	4	8	2	IIIc	2	2	1	46	1	death；36	1	36		
[illegible]	3	3	9	2	IIIc	2	2		51	1	loss	0			
[illegible]	0	4	0	2	IIIc	2	2	2	59	2	loss	0			
[illegible]	2	3	6	2	IIIC	2	2	2	60	2	loss	0			
[illegible]	2	3	6	2	IIIc	2	2	1	55	2	70	0	70		
[illegible]	3	2	6	2	IIIC	2	2	1	58	2	34	0	34		
[illegible]	3	2	6	2	IIIc	2	2	1	49	1	78	0	78		
[illegible]	2	3	6	2	IIIc	2	2		53	1	loss	0			
[illegible]	3	2	6	2	IIIc	2	2		71	2	loss	0			
[illegible]	2	3	6	2	IIIC	2	2	1	52	1	43	0	43		
[illegible]	2	3	6	2	IIIC	2	2	2	39	1	36	0	36		
[illegible]	2	3	6	2	IIIC	2	2	2	54	1	44	0	44		
[illegible]	3	2	6	2	IIIC	2	2	2	42	1	34	0	34		
[illegible]	2	4	8	2	IIIc	2	1	1	38	1	loss	0			
[illegible]	3	3	9	2	IIIc	2	2	2	62	2	39	0	39		
[illegible]	3	3	9	2	IIIC	2	2	2	50	1	32	0	32		
[illegible]	3	3	9	2	IIIC	2	2	1	48	1	loss	0			
[illegible]	3	3	9	2	IIIC	2	2	2	51	1	43	0	43		

HIC-3

NC	COLOR（0-3）	AREA（0-4）	SCOI	Gro	FIG	FIG	Gra	Lymphnc	ag	Ag	Survival t	Dea	Tim
[illegible]	3	3	9	2	IIIC	2	2	2	51	1	43	0	43
[illegible]	3	3	9	2	IIIC	2	2	2	49	1	loss	0	
[illegible]	3	4	12	2	IIIC	2	2		48	1	43	0	43
[illegible]	2	3	6	2	IIIc	2	2		49	1	63	0	63
[illegible]	2	2	4	1	III	2	2	1	52	1	death： 23	1	23
[illegible]	2	2	4	1	IIIc	2	1		55	2	death： 77	1	77
[illegible]	2	2	4	1	IIIb	2	2	1	43	1	loss	0	
[illegible]	2	2	4	1	IV	2	2	2	57	2	79	0	79
[illegible]	1	4	4	1	III b	2	1	2	46	1	104	0	104
[illegible]	1	3	3	1	IV	2	2	2	53	1	32	1	32
[illegible]	2	1	2	1	IIIc	2	1	1	69	2	46	0	46
[illegible]	1	3	3	1	IIIC	2	2	2	58	2	37	0	37
[illegible]	2	2	4	1	IIIc	2	1	1	69	2	121	1	121
[illegible]	2	2	4	1	IIIc	2	1	1	55	2	106	0	106
[illegible]	2	2	4	1	IIIC	2	2	2	40	1	44	1	44
[illegible]	2	1	2	1	IV	2	2	2	49	1	90	0	90
[illegible]	3	4	12	2	IA	1	1	1	64	2	69	0	69
[illegible]	2	4	8	2	Ib	1	1		70	2	72	0	72
[illegible]	3	3	9	2	IIa	1	1	1	62	2	loss	0	
[illegible]	3	2	6	2	II	1	1	1	60	2	loss	0	
[illegible]	2	4	8	2	Ic	1	1	1	55	2	62	0	62
[illegible]	2	4	8	2	I	1	2	1	48	1	75	0	75
[illegible]	3	3	9	2	Ia	1	1	1	74	2	67	0	67
[illegible]	0	4	0	2	Ic	1	1		32	1	27	1	27
[illegible]	2	2	4	1	IIb	1	2	1	54	1	death： 45	1	45
[illegible]	2	2	4	1	IIc	1	2	1	74	2	66	0	66
[illegible]	2	1	2	1	I c	1	2	1	53	1	123	1	123
[illegible]	0	3	0	1	Ia	1	2	1	51	1	111	0	111
[illegible]	2	1	2	1	Ia	1	1	1	58	2	100	1	100
[illegible]	2	2	4	1	Ia	1	1	1	40	1	loss	0	
[illegible]	2	1	2	1	IA	1	1	1	35	1	43	1	43
[illegible]	1	4	4	1	Ic	1	2	1	47	1	83	0	83
[illegible]	1	3	3	1	Ic	1	1	1	45	1	loss	0	
[illegible]	0	4	0	1	Ic	1	2		63	2	85	0	85
[illegible]	2	2	4	1	IIA	1	2	1	65	2	32	1	32
[illegible]	2	2	4	1	IIc	1	1	1	54	1	loss	0	
[illegible]	2	1	2	1	Ib	1	2	1	56	2	49	1	79
[illegible]	0	4	0	1	Ic	1	1	1	58	2	46	1	46
[illegible]	2	1	2	1	Ic	1	1	1	61	2	86	0	86
[illegible]	2	2	4	1	I	1	1	1	74	2	58	0	58
[illegible]	2	2	4	1	I	1	2	1	44	1	76	1	76
42	2	2	4	1	Ic	1	1	1	52	1	loss	0	

MTT-1

OVCAR3	0.196	0.13	0.188	0.196	0.211	0.219	0.213	0.209	0.223	0.215
OVCAR3-L-1	0.222	0.208	0.199	0.206	0.209	0.214	0.21	0.204	0.212	0.22
OVCAR3-L-2	0.206	0.215	0.213	0.208	0.211	0.214	0.212	0.197	0.193	0.211

	0.39	0.385	0.374	0.371	0.373	0.371	0.385	0.395	0.393	0.397
OVCAR3	0.465	0.416	0.423	0.407	0.426	0.43	0.429	0.427	0.404	0.451
OVCAR3-L-1	0.36	0.405	0.374	0.379	0.377	0.384	0.379	0.389	0.408	0.427
OVCAR3-L-2	0.374	0.403	0.352	0.372	0.374	0.39	0.402	0.345	0.371	0.409

	0.563	0.522	0.507	0.528	0.527	0.522	0.52	0.544	0.525	0.534
OVCAR3	0.664	0.643	0.655	0.654	0.644	0.637	0.625	0.691	0.635	0.713
OVCAR3-L-1	0.434	0.471	0.449	0.475	0.473	0.483	0.461	0.496	0.51	0.511
OVCAR3-L-2	0.49	0.488	0.458	0.467	0.484	0.475	0.512	0.503	0.491	0.514

	0.714	0.692	0.654	0.712	0.697	0.7	0.7	0.699	0.701	0.7
OVCAR3	1.144	1.074	0.99	1.033	1.075	1.06	1.076	1.089	1.06	1.001
OVCAR3-L-1	0.671	0.571	0.608	0.649	0.696	0.715	0.675	0.577	0.674	0.658
OVCAR3-L-2	0.628	0.67	0.571	0.632	0.607	0.563	0.588	0.636	0.63	0.743

	0.838	0.818	0.839	0.816	0.819	0.829	0.847	0.809	0.812	0.841
OVCAR3	1.053	1.059	1.081	1.05	1.092	1.077	1.077	1.072	1.051	1.117
OVCAR3-L-1	0.756	0.77	0.721	0.684	0.725	0.738	0.744	0.753	0.747	0.797
OVCAR3-L-2	0.788	0.681	0.713	0.71	0.744	0.772	0.799	0.734	0.73	0.805

A2780	0.148	0.16	0.148	0.161	0.155	0.162	0.153	0.164	0.161	0.167
A2780-L-1	0.153	0.151	0.148	0.163	0.152	0.154	0.15	0.147	0.146	0.148
A2780-L-2	0.153	0.165	0.166	0.159	0.163	0.17	0.162	0.164	0.163	0.168

A2780	0.325	0.313	0.315	0.308	0.31	0.307	0.3	0.281	0.313	0.346
A2780-L-1	0.26	0.217	0.211	0.253	0.237	0.243	0.225	0.236	0.252	0.25
A2780-L-2	0.208	0.227	0.234	0.22	0.213	0.223	0.226	0.22	0.225	0.209

A2780	0.884	0.775	0.716	0.69	0.785	0.722	0.779	0.804	0.798	0.799
A2780-L-1	0.547	0.545	0.468	0.47	0.499	0.539	0.554	0.53	0.517	0.556
A2780-L-2	0.557	0.54	0.455	0.538	0.502	0.524	0.518	0.504	0.544	0.601

A2780	0.908	0.93	0.954	0.931	0.95	0.981	0.936	0.946	0.935	0.955
A2780-L-1	0.645	0.661	0.613	0.582	0.618	0.619	0.634	0.643	0.628	0.677
A2780-L-2	0.673	0.585	0.624	0.6	0.628	0.646	0.668	0.628	0.621	0.675

A2780	1.233	1.234	1.281	1.26	1.274	1.218	1.244	1.261	1.244	1.267
A2780-L-1	0.887.	0.912	0.859	0.861	0.842	0.853	0.894	0.891	0.889	0.932
A2780-L-2	0.9	0.804	0.848	0.837	0.828	0.861	0.884	0.845	0.835	0.845

MTT-2

D1										
A2780	0.184	0.183	0.193	0.187	0.183	0.191	0.193	0.183	0.198	0.188
A2780-MOCK	0.181	0.179	0.175	0.189	0.171	0.189	0.187	0.185	0.184	0.181
A2780-H	0.194	0.2	0.188	0.183	0.188	0.185	0.2	0.187	0.194	0.187
D2										
A2780	0.317	0.3	0.304	0.279	0.272	0.283	0.293	0.296	0.292	0.292
A2780-MOCK	0.308	0.296	0.316	0.321	0.209	0.32	0.313	0.359	0.317	0.363
A2780-H	0.334	0.351	0.325	0.321	0.333	0.322	0.335	0.328	0.312	0.356
D3										
A2780	0.48	0.463	0.467	0.446	0.449	0.485	0.441	0.454	0.464	0.491
A2780-MOCK	0.494	0.471	0.493	0.503	0.487	0.473	0.5	0.44	0.466	0.499
A2780-H	0.635	0.609	0.561	0.603	0.616	0.595	0.615	0.647	0.654	0.664
D4										
A2780	0.917	0.949	0.89	0.932	0.946	0.918	0.974	1.012	0.974	0.994
A2780-MOCK	0.932	0.889	1.041	1.03	0.975	0.993	1.045	1.062	0.992	1.144
A2780-H	1.292	1.204	1.198	1.195	1.279	1.318	1.34	1.374	1.371	1.356
D5										
A2780	0.922	0.861	0.886	0.924	0.944	0.917	0.97	1.008	0.961	0.992
A2780-MOCK	0.927	0.891	1.016	1.018	0.967	0.987	1.042	1.057	0.987	1.141
A2780-H	1.297	1.216	1.199	1.193	1.279	1.316	1.337	1.369	1.373	1.359

D1										
OV	0.165	0.157	0.159	0.167	0.174	0.153	0.147	0.188	0.173	0.178
OV-MOCK	0.183	0.168	0.163	0.183	0.186	0.17	0.152	0.175	0.196	0.223
OV-H	0.204	0.195	0.185	0.183	0.172	0.167	0.163	0.177	0.159	0.19
D2										
OV	0.317	0.3	0.304	0.279	0.272	0.283	0.293	0.296	0.292	0.292
OV-MOCK	0.308	0.296	0.316	0.321	0.209	0.32	0.313	0.359	0.317	0.363
OV-H	0.334	0.351	0.325	0.321	0.333	0.322	0.335	0.328	0.312	0.356
D3										
OV	0.43	0.373	0.373	0.372	0.387	0.411	0.391	0.402	0.382	0.391
OV-MOCK	0.406	0.398	0.419	0.416	0.444	0.444	0.435	0.411	0.456	0.468
OV-H	0.443	0.454	0.394	0.393	0.394	0.401	0.398	0.399	0.421	0.429
D4										
OV	0.48	0.463	0.467	0.446	0.449	0.485	0.441	0.454	0.464	0.491
OV-MOCK	0.494	0.471	0.493	0.503	0.487	0.473	0.5	0.44	0.466	0.499
OV-H	0.635	0.609	0.561	0.603	0.616	0.595	0.615	0.647	0.654	0.664
D5										
OV	0.917	0.949	0.89	0.932	0.946	0.918	0.974	1.012	0.974	0.994
OV-MOCK	0.932	0.889	1.041	1.03	0.975	0.993	1.045	1.062	0.992	1.144
OV-H	1.292	1.204	1.198	1.195	1.279	1.318	1.34	1.374	1.371	1.356
